# Parasympathetic tonus in type 2 diabetes and pre-diabetes and its clinical implications

**DOI:** 10.1038/s41598-022-22675-2

**Published:** 2022-10-26

**Authors:** Rakin Hadad, Sarah F. Akobe, Philip Weber, Christoffer V. Madsen, Bjørn Strøier Larsen, Sten Madsbad, Olav W. Nielsen, Maria Helena Dominguez, Steen B. Haugaard, Ahmad Sajadieh

**Affiliations:** 1 Department of Cardiology, Copenhagen University Hospital of Bispebjerg, Bispebjerg Bakke 23, 2400 Copenhagen NV, Denmark; 2 Department of Endocrinology, Copenhagen University Hospital of Bispebjerg, Bispebjerg Bakke 23, 2400 Copenhagen NV, Denmark; 3 Department of Endocrinology, Copenhagen University of Hvidovre, Kettegård Alle 30, 2650 Hvidovre, Denmark; 4 Institute of Clinical Medicine, Faculty of Health and Medical Sciences, University of Copenhagen, Copenhagen, Denmark

**Keywords:** Cardiology, Endocrinology, Pathogenesis

## Abstract

Autonomic imbalance reflected by higher resting heart rate and reduced parasympathetic tone may be driven by low-grade inflammation (LGI) and impaired glycemic control in type 2 diabetes mellitus (T2DM) and pre-diabetes. We examined the interaction of parasympathetic components of heart rate variability (HRV), variables of LGI, and glucose metabolism in people with T2DM, pre-diabetes, and normal glucose metabolism (NGM). We recorded HRV by Holter (48 h) in 633 community-dwelling people of whom T2DM n = 131, pre-diabetes n = 372, and NGM n = 130 and mean HbA1c of 7.2, 6.0 and 5.3%, respectively. Age was 55–75 years and all were without known cardiovascular disease except from hypertension. Fasting plasma glucose, fasting insulin, HOMA-IR, HbA1c and LGI (CRP, Interleukin-18 (IL-18), and white blood cells) were measured. Root-mean-square-of-normal-to-normal-beats (RMSSD), and proportion of normal-to-normal complexes differing by more than 50 ms (pNN50) are accepted measures of parasympathetic activity. In univariate analyses, RMSSD and pNN50 were significantly inversely correlated with level of HbA1c and CRP among people with T2DM and pre-diabetes, but not among NGM**.** RMSSD and pNN50 remained significantly inversely associated with level of HbA1c after adjusting for age, sex, smoking, and BMI among people with T2DM (β = − 0.22) and pre-diabetes (β = − 0.11); adjustment for LGI, HOMA-IR, and FPG did not attenuate these associations. In backward elimination models, age and level of HbA1c remained associated with RMSSD and pNN50. In people with well controlled diabetes and pre-diabetes, a lower parasympathetic activity was more related to age and HbA1c than to markers of LGI. Thus, this study shows that the driver of parasympathetic tonus may be more the level of glycemic control than inflammation in people with prediabetes and well controlled diabetes.

## Introduction

Type 2 diabetes mellitus (T2DM) is a major health burden in the western world^1^ and is associated with complications affecting major organ systems^2–4^, including autonomic dysfunction and cardiac autonomic neuropathy (CAN). The prevalence of CAN spans from 15% among newly diagnosed T2DM^5^ to 90% among people with a long history of type 1 diabetes^6^. The function of the autonomic nervous system can be assessed by measuring the heart rate variability (HRV), which is the mathematical expression of the fluctuations of heart rate regulated predominantly by the autonomic nervous system^7^. Signs of CAN include resting tachycardia, exercise intolerance, abnormal blood pressure regulation, and orthostatic hypotension^8^. It was believed that CAN related to the onset of diabetes, but autonomic dysfunction is prevalent already in the pre-diabetes state and progresses during manifest diabetes^9^. A recent systematic review concluded that the prevalence of CAN ranged from 9 to 39% among people with pre-diabetes^10^. Autonomic imbalance, as indicated by a reduced HRV, is a strong marker of vascular disease, especially microvascular disease^11^, but may also play a central role in the association between level of glycated hemoglobin (HbA1c) and subclinical atherosclerosis^12^.

The causal pathophysiology of T2DM is insulin resistance and β-cell dysfunction^13^. In the past 20 years, the most prevalent hypothesis for β-cell dysfunction relates to glucolipotoxicity and subclinical systemic low-grade inflammation (LGI) from unhealthy lifestyle factors including obesity. The ARIC cohort suggested elevated inflammatory markers as a central driver of glycemia leading to diabetes^14^ as confirmed in subsequent studies^15,16^. Hyperglycemia is a significant contributor to the development of diabetic microvascular complications^17^. Whether to focus on improving glycemic control or LGI in the treatment of pre-diabetes and T2DM has been addressed previously^18^, although addressing one of the factors with modern antidiabetic medication often also improve the other^19,20^.

This study investigates the association between parasympathetic tonus versus markers of glucose metabolism and LGI, in people with T2DM, pre-diabetes, and normal glucose metabolism (NGM).

## Methods

**Figure 1:**
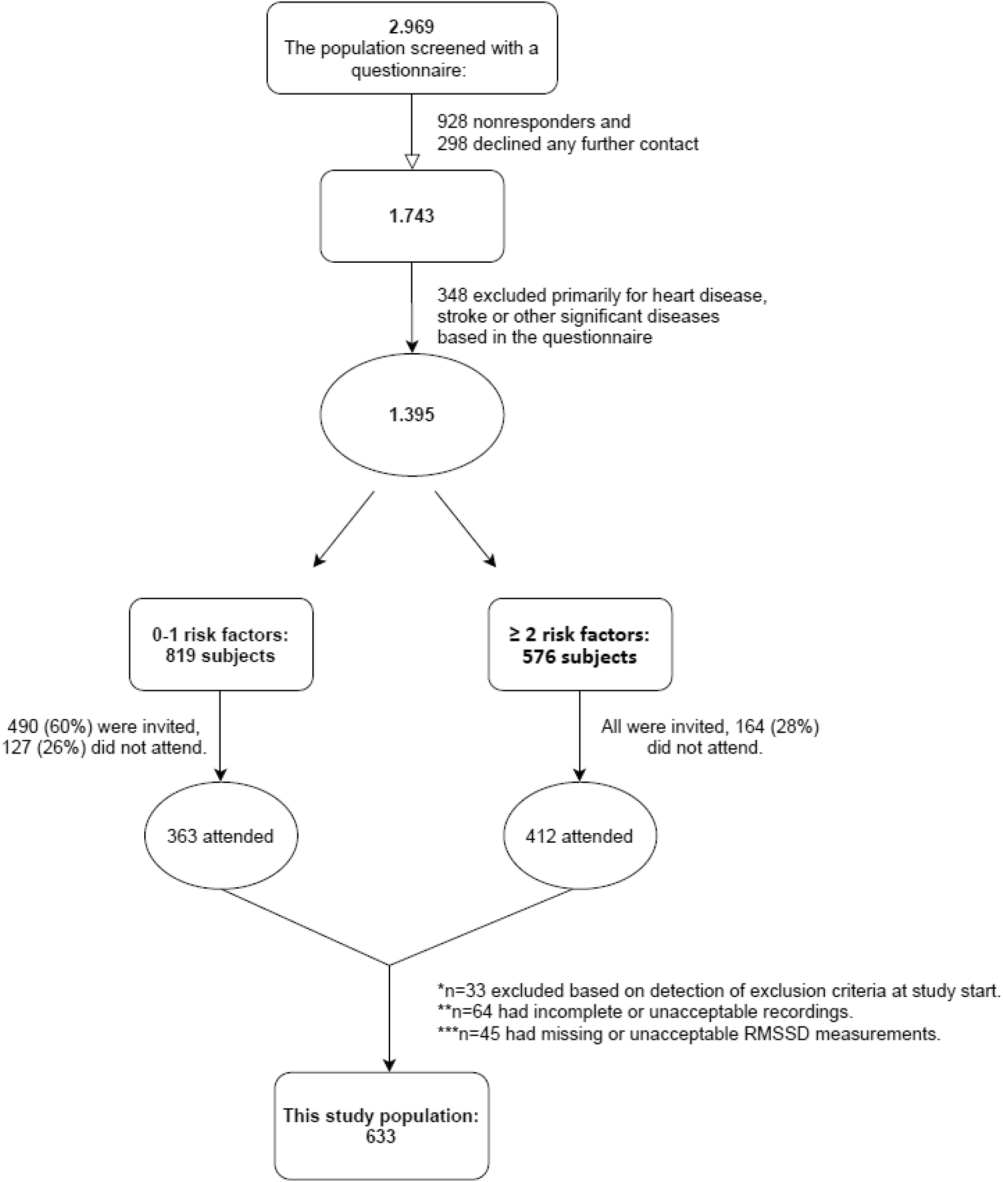
Flow diagram demonstrating the study population at various exclusion steps.

This study is part of The Copenhagen Holter Study, a population-based cohort study, conducted between April 1998 and June 2000. Details of the selection procedures have been described previously^21,22^. The study aimed to evaluate 48-h Holter monitoring recordings in relation to risk assessment of middle-aged and older men and women without apparent heart disease. All participants were included from two well-defined postal regions of Copenhagen, Denmark. All men aged 55 and men and women aged 60, 65, 70, and 75 (n = 2969) were asked to fill in a questionnaire on cardiovascular risk factors, medical history, and medication use. All subjects were ranked according to numbers of the following self-reported risk factors, including hypertension, diabetes mellitus, smoking habits, familial history of cardiac diseases (sudden death or acute myocardial infarction), obesity (body mass index ≥ 30 kg/m^2^), or known hypercholesterolemia. Exclusion criteria were: (1) manifest ischemic heart disease, e.g., a history of acute myocardial infarction, coronary revascularization, and angina pectoris; (2) other manifest cardiac diseases, e.g., congestive heart failure, valvular heart disease including atrial fibrillation or medical treatment for any heart disease; (3) a history of stroke; (4) cancer; (5) other significant or life-threatening conditions, e.g., cirrhosis hepatitis, renal insufficiency requiring dialysis, and chronic pulmonary disease requiring home oxygen therapy; (6) technical reasons resulting in interruption of recording or poor quality of the recording. Technical reasons for exclusion included unacceptable or incomplete Holter recordings as well as findings of interrupting arrhythmias during the recording. Subjects with moderately increased atrial or ventricular ectopy were not excluded. However, subjects with excessive arrhythmias (premature atrial contractions and premature ventricular contractions) were excluded. 633 participants fulfilled inclusion criteria and obtained acceptable Holter monitoring (Fig. 1).

Forty-eight-hour Holter monitoring was performed by two-channel “Space Labs” tape recorders (9025, SpaceLabs Inc., Redwood, Wa, USA). FT3000 Medical Analysis and Review Station were applied. All Holter analyses were evaluated by trained technicians at the Holter Laboratory of the Copenhagen University Hospital, Hvidovre. The personal were blinded to patient data. A 24-h period was selected for analysis, i.e. from the second to the 25th hour, to avoid initial noise.

We measured time-domain components of HRV. SDNN was defined as standard deviation (SD) for the mean value of normal-to-normal (NN) complexes and reflects the total variability. RMSSD is defined as the root mean square of successive NN interval differences and is the primary time domain measure used to estimate the vagal changes in HRV^23^. pNN50 is defined as the proportion of NN complexed that differ by more than 50 ms was also used as a marker of parasympathetic activity. From the Holter monitoring 24-heart rate was derived from mean NN; 60.000/mean NN = 24-h average heart rate.

Diabetes mellitus was diagnosed by fulfilling at least one of the following criteria: Known T2DM, fasting plasma glucose (FPG) ≥ 7.0 mmol/mol, or HbA1c ≥ 6.5% (48 mmol/mol). Pre-diabetes was defined as HbA1c between 5.7 and 6.4% (39–47 mmol/mol). Diabetes and pre-diabetes status were determined at baseline before Holter monitoring. Testing was done between 7:00 and 10:00 a.m. after a fasting period of at least 8 hours. Venous blood was collected in evacuated glass tubes. Separation of plasma and serum was done immediately after collection by centrifuging at 2000 G for 15 min. All samples were stored at -70 °C. Analysis of plasma glucose was done using a Hitachi 7170 (Tokyo, Japan) automated analyzer. Insulin resistance was based on the model assessment on insulin resistance index (HOMA-IR), which corresponds well to insulin measurements through clamp-derived methods^24^.

As markers for low-grade inflammation, we selected high sensitivity C-reactive protein (hs-CRP), interleukin-18 (IL-18), and white blood cell count (WBC). hs-CRP and WBC are well-known markers of inflammation and sub-inflammation^15,16^. IL-18 has been associated with chronic LGI and found elevated in people with type 2 diabetes and metabolic syndrome^25–27^. Immunofluorescence technique by “Kryptor” BRAHMS (Saint-Ouen, France) was used to measure hs-CRP. IL-18 was measured through multiplexed sandwich immunoassays based on flowmetric Luminex (Luminex Corp., Austin, TX, USA). Physical activity was dichotomized in two groups: Group 1 included participants with an almost sedentary lifestyle or only light physical activity of 2–4 h per week. Group 2 included participants with moderate to a high level of physical activity or training of at least 4 h per week.

### Statistical analyses

All statistical analyses were performed using SAS Studio statistical software version 3.8. Mean and standard deviations are presented for continuous normally distributed variables. Otherwise, median values with interquartile ranges are shown. Test for normality was done graphically by histograms as well as Shapiro–Wilk test. A p-value > 0.05 was considered evidence for normal distribution. Baseline variables between participants with diabetes, pre-diabetes, and NGM were compared by student’s t-test, Kruskal–Wallis test, Spearman’s rank correlation, or Pearson chi-square test. We used multivariable linear regression to assess the association between glycated hemoglobin, LGI, and HRV. In the model, we adjusted for age, sex, body mass index (BMI), smoking, systolic blood pressure (SBP), HOMA-IR, fasting plasma glucose, HbA1c, and LGI. A two-sided p-value < 0.05 was considered statistically significant. We performed collinearity diagnostics to evaluate multicollinearity between variables of interest. According to literature, a condition index of ≥ 30 indicates problems with collinearity among variables^28^. If a variable showed a condition index of ≥ 30, we looked at the variance proportions between variables to identify the source of collinearity. If the variables showed variance proportions of ≥ 0.9 between more than one variable, the variable was removed from the model. To determine the strongest variables in the linear regression models we performed a backward elimination model with all variables included and a cut-off value of p = 0.20 to stay in the model.

### Ethics approval and consent to participate

All subjects in this study were informed of the study and written informed consent was obtained from all subjects. The regional ethical committee (“De Videnskabsetiske Komiteer for Region Hovedstaden”) approved the study and the Declaration of Helsinki was followed.

## Results

**Figure 2:**
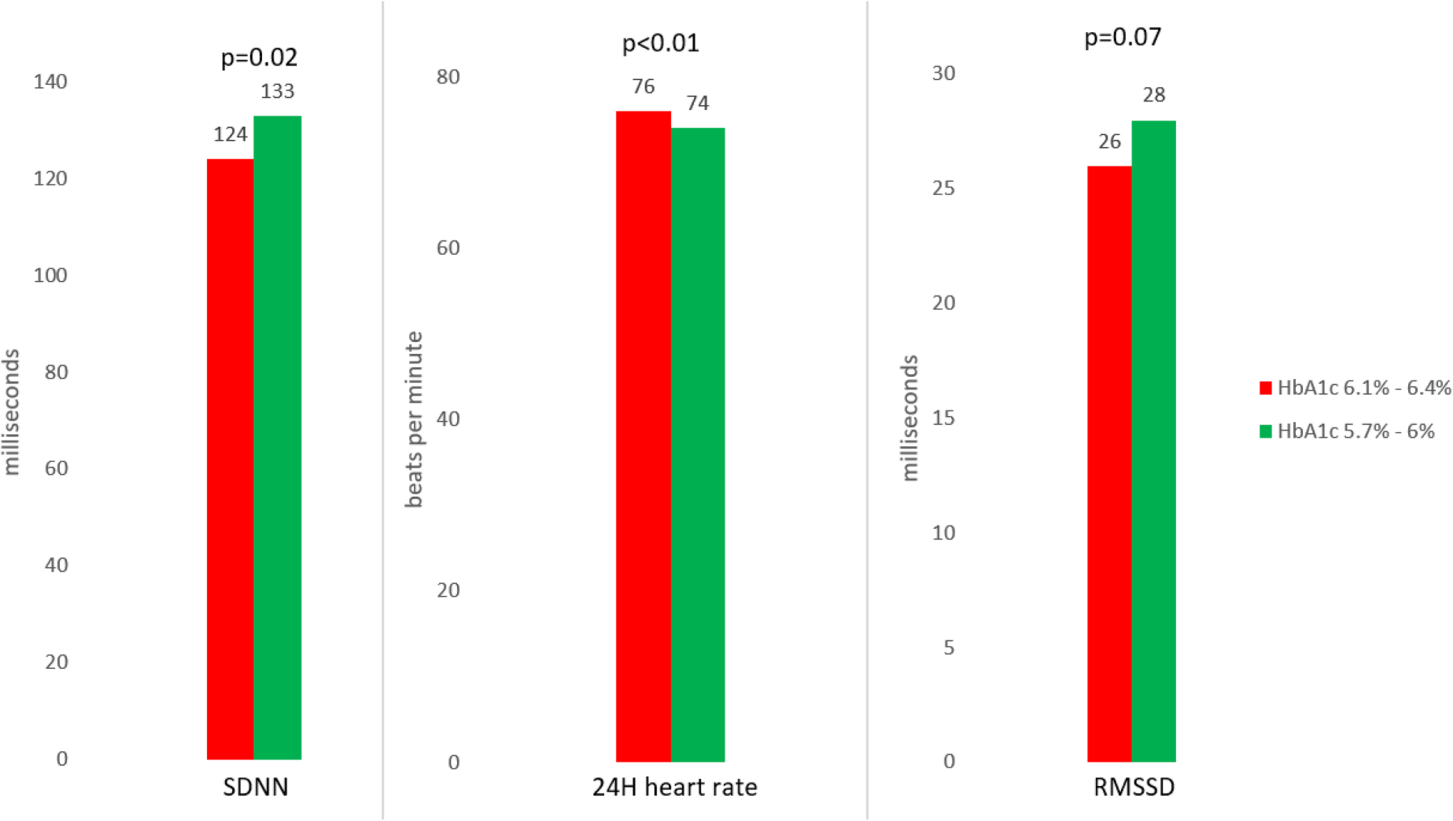
Heart rate variability and heart rate among participants with pre-diabetes stratified for glycated hemoglobin (HbA1c): HbA1c 5.7–6% and HbA1c 6.1–6.4%. *SDNN* Standard deviation of normal-normal beats, *RMSSD* Square root of the mean squared differences of successive NN intervals.

In this study, 131 participants met the diagnostic criteria for T2DM. Of the 131 participants with diabetes, 43 had known diabetes, 78 participants had a HbA1c ≥ 6.5% and 10 had FPG ≥ 7.0 µmol/l. Participants with diabetes were treated medically by Metformin or Sulfonylurea. Three-hundred-seventy-two participants met the criteria for pre-diabetes, and 130 participants displayed NGM. The mean value of HbA1c was 5.3% for NGM, 6.0% among pre-diabetes, and 7.2% among T2DM. Baseline characteristic are shown in Table 1. Participants with T2DM had higher levels of triglycerides, inflammatory markers and lower levels of SDNN and RMSSD. In participants with pre-diabetes a higher HbA1c was associated with higher 24-h average heart rate and lower SDNN (Fig. 2).

**Table 1:** Baseline characteristics of subjects with pre-diabetes and diabetes compared to subjects with normal glucose tolerance (NGM).

Baseline variables	All subjects (n = 633)	Subjects with NGM (n = 130)	Subjects with pre-diabetes (n = 372)	Subjects with diabetes (n = 131)
**Participants characteristic**				
Sex (female)	276 (44%)	57 (44%)	174 (47%)	45 (34%)
Age (years)	64 (6.8)	64 (6.5)	64 (6.7)	65 (7)
Body mass index (kg/m^2^)	26 (4.2)	26 (4.0)	26 (4.2)	27(4.3)
Current smoker (yes)	338 (53%)	92 (71%)	181 (49%)^‡^	65 (50%)^‡^
**Blood pressure (mmHg)**				
Systolic	157 (24)	154 (23)	156 (26)	161 (21)^†^
Diastolic	91 (11)	90 (11)	90 (11)	93 (10)^†^
**Blood test**				
Hemoglobin (mmol/L)	8.9 (0.69)	8.7 (0.72)	8.9 (0.67)	9.0 (0.68)^‡^
Fasting plasma glucose (mmol/L)	5.8 (1.7)	5.3 (0.6)	5.4 (0.6)^†^	7.5 (3.1)^‡^
HbA1c (%)	6.2 (0.8)	5.3 (0.4)	6.0 (0.2)^‡^	7.2 (1.3)^‡^
HbA1c (mmol/mol)	44 (9)	34 (5)	42 (4)^‡^	55 (14)^‡^
Insulin (pM)	38 (22–59)	26 (14–43)	38 (23–57)^‡^	48 (31–80)^‡^
HOMA-IR	0.97 (0.53–1.63)	0.63 (0.36–1.06)	0.91 (0.55–1.48)^‡^	1.56 (0.97–2.80)^‡^
Plasma creatinine (µmol/mL)	93 (19)	92 (15)	91 (13)	98 (32)
Total cholesterol (mmol/L)	6.1 (1.0)	5.9 (1.0)	6.1 (1.1)^†^	6.1 (1.0)
Low density lipoprotein (mmol/L)	3.9 (0.95)	3.7 (0.9)	3.9 (0.95)^‡^	3.9 (1.0)†
Triglycerides (mmol/L)	1.25 (0.92–1.83)	1.13 (0.89–1.63)	1.20 (0.88–1.73)	1.54 (1.18–2.33)^‡^
NT-proBNP (pmol/L)	6.88 (3.59–13.1)	7.93 (4.47–14.7)	6.34 (3.46–12.4)	6.7 (3.61–13.8)
hs-CRP (µg/L)	2.46 (1.14–4.61)	2.15 (0.91–4.15)	2.32 (1.14–4.46)	3.0 (1.75–6.0)‡
IL-18 (pg(mL))	541 (401–728)	505 (399–692)	523 (387–727)	614 (435–820)†
White blood cell count (10^9^/L)	6.5 (1.9)	6.1 (1.9)	6.4 (1.8)†	7.0 (2.0)‡
**Medication**				
Anti-diabetes medication (n)	42 (6.4%)	NA	NA	42 (32%)
Use of aspirin (n)	92 (15%)	21 (16%)	48 (13%)	23 (18%)
Use of statin (n)	14 (2.2%)	1 (0.8%)	10 (2.7%)	3 (2.3%)
Use of anti-hypertensive (n)	164 (26%)	34 (26%)	78 (21%)	52 (40%)†
**Heart rate and heart rate variability**				
24-h heart rate (bpm)*	76 (9)	76 (10)	75 (9)	77 (8)
24-h SDNN (ms)	125 (35)	125 (38)	128 (33)	115 (34)†
24-h RMSSD (ms)	22 (17–31)	22 (17–32)	23 (18–32)	20 (15–29)†
24-h pNN50 (%)	2.3 (0.8–5.6)	1.95 (0.7–5.8)	2.5 (0.9–6.2)	1.7 (0.7–4.4)

Results are presented as mean ± standard deviation (SD), median (Q1–Q3), or n (%).

Antihypertensive medicine indicates the use of one of the following: β-blockers, calcium antagonists, ACE inhibitors, or diuretics.

*24-h hear rate has been derived from MEAN_NN_: 60.000/MEAN_NN_ = average heart rate.

^†^P-value < 0.05. P-value indicates difference from subjects with NGM.

^‡^P-value < 0.01. P-value indicates difference from subjects with NGM.

### Measures of autonomic nervous balance and their associations with HbA1c and LGI

Table 2 shows the univariate associations between parasympathetic components of HRV and variables related to LGI and glucose metabolism. RMSSD and pNN50 were inversely correlated with levels of HbA1c in the whole study population, T2DM and pre-diabetes participants, but not among NGM participants. RMSSD was also inversely correlated with hs-CRP in the whole study population but not in sub-group analyses (Table 2). Other variables of LGI were associated with SDNN and 24-h heart rate. These associations were greater among participants with T2DM and pre-diabetes compared to NGM. LGI was also associated with HOMA-IR in the whole study population, but was only observed in the sub-group of participants with pre-diabetes.

**Table 2:** Measures of heart rate, heart rate variability, glycated hemoglobin (HbA1c), insulins resistance (HOMA-IR) and different inflammation markers of interest and their association.

	HbA1c	FPG	Insulin	HOMA-IR	*hs*-CRP	IL-18	WBC
**Whole study population (N = 633)**							
RMSSD	− 0.13^†^	− 0.06	− 0.02	− 0.05	− 0.08*	− 0.02	− 0.06
pNN50	− 0.11^†^	− 0.06	− 0.02	− 0.04	− 0.07	− 0.03	− 0.07
SDNN	− 0.10*	− 0.06	− 0.03	− 0.05	− 0.17^†^	− 0.01	− 0.17^†^
24H HR	0.11^†^	0.09*	0.05	0.06	0.22^†^	0.05	0.19^‡^
HOMA-IR	0.25^†^	0.51^†^	0.97^†^	NA	0.11*	0.11^†^	0.13^†^
**Participants with diabetes (N = 131)**							
RMSSD	− 0.21^†^	− 0.05	0.03	− 0.02	− 0.11	− 0.05	− 0.10
pNN50	− 0.19*	− 0.01	0.08	0.03	− 0.09	− 0.10	− 0.13
SDNN	− 0.09	0.06	0.05	0.02	− 0.30^†^	− 0.16	− 0.24^†^
24H HR	0.11	0.001	− 0.03	− 0.01	0.34^†^	− 0.08	0.19*
HOMA-IR	0.11	0.53^†^	0.94^†^	NA	0.06	0.05	− 0.05
**Participants with pre**− **diabetes (N = 372)**							
RMSSD	− 0.11*	− 0.05	− 0.03	− 0.04	− 0.05	− 0.002	− 0.05
pNN50	− 0.11*	− 0.06	− 0.06	− 0.07	− 0.05	− 0.01	− 0.04
SDNN	− 0.09	− 0.05	− 0.10*	− 0.10*	− 0.09	− 0.05	− 0.13^†^
24H HR	0.13^†^	0.07	0.11*	0.11*	0.20^†^	0.13*	0.21^†^
HOMA-IR	0.05	0.48^†^	0.99^†^	NA	0.09	0.12*	0.15^†^
**Participants with normal glucose metabolism (N = 130)**							
RMSSD	− 0.04	0.02	0.05	0.05	− 0.09	0.06	0.005
pNN50	− 0.04	0.05	0.08	0.08	− 0.09	0.04	− 0.03
SDNN	− 0.009	− 0.06	0.20*	0.20*	− 0.20*	0.02	− 0.17
24H HR	0.12	0.13	− 0.08	− 0.07	0.15	− 0.05	0.15
HOMA-IR	− 0.1	0.16*	0.99^†^	NA	0.01	− 0.02	− 0.01

Results are presented as correlation coefficient (r).

*hs-CRP* High sensitivity C-reactive protein, *IL18* Interleukin-18, *WBC* White blood cell count, *FPG* Fasting plasma glucose, *HOMA-IR* Homeostatic model assessment for insulin resistance, *SDNN* Standard deviation of normal-normal complexes, *RMSSD* Root mean square of successive normal-normal interval differences, *pNN50* proportion of NN intervals which differs by more than 50 ms, *24H HR* 24-h heart rate. Derived from MEAN_NN_: MEAN_NN_/60.000 = Average HR.

*p < 0.05.

^†^p < 0.01.

In multivariable analyses, RMSSD and pNN50 remained associated with levels of HbA1c after adjusting for age, sex, smoking habits, and BMI (model 1) in participants with T2DM and pre-diabetes (Table 3). Adjustment for markers of inflammation (hs-CRP, IL-18, and WBC), HOMA-IR, and FPG did not attenuate associations (Table 3, model 2), and neither did the inclusion of physical activity, antihypertensive medication, plasma creatinine, or lipid profiles into the models (data not shown). SDNN as a mixed sympathetic/parasympathetic variable was associated with HbA1c in the total study population but not in sub-group analyses.

**Table 3:** Multivariate linear regression models showing the associations between glycated hemoglobin (HbA1c) and variables of heart rate and heart rate variability in people with normal glucose metabolism, pre-diabetes, and type 2 diabetes.

24-Hour RMSSD	HbA1c outcome in diabetes			HbA1c outcome in pre-diabetes			HbA1c outcome in normal glucose metabolism		
	*β*	*t*	*p*	*β*	*t*	*p*	*β*	*t*	*p*
Model 1	− 0.22	− 2.61	0.01	− 0.11	− 2.11	0.03	0.03	0.26	0.80
Model 2	− 0.38	− 2.54	0.01	− 0.11	− 2.02	0.04	0.05	0.45	0.65

24-Hour pNN50	Diabetes			Pre-diabetes			Normal glucose metabolism		
	*β*	*t*	*p*	*β*	*t*	*p*	*β*	*t*	*p*
Model 1	− 0.22	− 2.55	0.01	− 0.11	− 2.03	0.04	0.02	0.23	0.82
Model 2	− 0.40	− 2.62	0.01	− 0.11	− 1.98	0.048	0.05	0.45	0.66

24-Hour SDNN	Diabetes			Pre-diabetes			Normal glucose metabolism		
	*β*	*t*	*p*	*β*	*t*	*p*	*β*	*t*	*p*
Model 1	− 0.12	− 1.39	0.17	− 0.06	− 1.16	0.25	0.07	0.74	0.46
Model 2	− 0.24	− 1.64	0.10	− 0.05	− 0.84	0.40	0.08	0.85	0.40

24-Hour HR	Diabetes			Pre-diabetes			Normal glucose metabolism		
	*β*	*t*	*p*	*β*	*t*	*p*	*β*	*t*	*p*
Model 1	0.11	1.36	0.17	0.13	2.49	0.01	− 0.02	− 0.16	0.87
Model 2	0.16	1.13	0.26	0.11	2.05	0.03	− 0.01	− 0.09	0.93

Results are presented as standardized regression coefficient β representing standard deviation (SD) change of HRV or HR for 1 SD change in HgbA1c (1 SD is equivalent to 0.4% HbA1c among NGM, 0.2% HbA1c among pre-diabetes, and 1.3% HbA1c among T2DM).

Model 1: Adjusted for age, sex, body mass index (BMI), and smoking.

Model 2: Further adjustment for homeostatic model assessment for insulin resistance (HOMA-IR), fasting plasma glucose, high-sensitive C-reactive protein (hs-CRP), and Interleukin-18 (IL-18).

Logarithmic value of RMSSD, IL-18, and hs-CRP is used, as these are not normally distributed.

24-Hour HR is derived from 24-h MEAN_NN_ (60.000/MEAN_NN_ = average 24-h HR in beats/min).

In collinearity analysis IL-18, hs-CRP, and WBC showed a condition index of ≥ 30^33,41^, but none of the variables showed a proportion variation of ≥ 0.90 between any other variables. We, therefore, used all variables in the full model in the multivariate analyses.

To assess which variables, have the highest impact we used backward elimination. In a model with RMSSD or pNN50 as dependent variables and LGI markers, glucose metabolic parameters (HbA1c, FPG, HOMA-IR, and insulin), triglycerides, creatinine, age, sex, BMI, smoking habits, and physical activity, as independent variables, only age and HbA1c stayed significantly associated with RMSSD and pNN50. This was observed in the total study population and went stronger through the gradient of glucose metabolism i.e. showing higher associations in the pre-diabetes and T2DM subgroups and no association among the NGM subgroup and (Table 4).

**Table 4:** Backward elimination model evaluating variables best associated with parasympathetic components of heart rate variability (RMSSD and pNN50).

	Age	HbA1c
**Whole population n = 633**		
RMSSD	0.030^‡^	0.031^‡^
pNN50	0.010*	0.027^‡^
**Diabetes mellitus n = 131**		
RMSSD	0.071^‡^	0.052^‡^
pNN50	0.064^‡^	0.050^‡^
**Pre-diabetes n = 372**		
RMSSD	0.021^‡^	0.015*
pNN50	0.006	0.012*
**Normal glucose metabolism n = 130**		
RMSSD	0.037*	0.005
pNN50	0.013	0.008

Results of the backward elimination selection regression presented as partial R^2^ for the variables. Only age, sex, and HbA1c stayed significant. Other variables; LGI markers, glucose metabolism variables (FPG, HOMA-IR, and insulin), triglycerides, creatinine, sex, BMI, smoking habits, and physical activity remained insignificant in all models except for creatinine in the subgroup of normal glucose metabolism.

*RMSSD* Root mean square of successive normal-normal interval differences, *pNN50* Proportion of NN intervals which differs by more than 50 ms. Logarithmic values of RMSSD, pNN50.

Physical activity was dichotomized in two groups: Group 1 included participants with an almost sedentary lifestyle or only light physical activity of 2–4 h per week. Group 2 included participants with moderate to high levels of physical activity or training of at least 4 h per week.

*p < 0.05.

^‡^p < 0.01.

## Discussion

### Hyperglycemia and reduced parasympathetic tone

In this cross-sectional study, we show that level of HbA1c and age are independently associated with parasympathetic tonus in people with pre-diabetes and well controlled diabetes. Postprandial hyperglycemia is the major contributor to higher HbA1c among people with well controlled T2DM, as shown in individuals with HbA1c lower than 7%^29^. Our data suggest that impaired parasympathetic tone is present in prediabetes and this impairment worsens as the disease progress to diabetes. This is in agreement with previous studies that find associations between glycemic fluctuations, dyslipidemia and associated markers of insulin resistance with parasympathetic imbalance^30–32^. In the same way, glycemic control with insulin improves sympatho-vagal tone type 2 diabetes^33^. While the r^2^ for the associations between RMSSD and HbA1c are seemingly small, it does not necessarily translate to a small effect since the cumulative effect over time could be significant.

It is known that age is inversely correlated with HRV in both men and women, which might be due to decreased vagal tone with higher age^34^. The decrease of HRV among aging people is more significant among people with cardiovascular diseases and other comorbidities compared to healthy individuals^35^. In our study the associations between age and RMSSD are quite similar in size as those related to HbA1c, implying a similar effect size on CAN from age and elevated HbA1c. It is possible a cumulative effect of HbA1c and age is present, worsening the effects of CAN.

Although a cause-effect relationship is not established, the present study leads support to the notion that both age and hyperglycemia are important regulators of the parasympathetic tone. High pulse rate and LGI are aspects of diabetes and pre-diabetes^16^, but the trigger to increased heart rate and reduced HRV is not elucidated. We suggest that postprandial hyperglycemia may play a significant role in impairment of heart rate and HRV. The direct effects of glucose excursions on central and peripheral nerve function have been described showing that both hyperglycemia and hypoglycemia have a detrimental effect on the peripheral nerve system as well as the central nerve system^36,37^. Geijselaers et al. showed that diabetes associated to worse performance in processing speed, memory, and executive function, and attention, which was primarily driven by hyperglycemia^38^. A reduced parasympathetic tone has two significant consequences: (a) aggravation of the inflammatory response, the inflammatory reflex as properly shown by Tracey et al.^39^, and (b) an increased longstanding heart rate which is a hallmark of diabetes and a risk factor for cardiovascular disease^40^.

#### Parasympathetic tone and the inflammatory response

The associations between the inflammatory system, including the reticuloendothelial and lymphatic system, and parasympathetic vagal activity are well documented^41,42^, e.g. an inflammatory reflex system exists by which the autonomic nervous system can communicate with the immune system and vice versa. Thus, a bidirectional communication is established, by which neurons in the central nervous system synthesize and express cytokines and stimulate other organs to do so in response to inflammation in peripheral tissue. The inflammatory response activates the cholinergic anti-inflammatory pathway in which cytokine synthesis is inhibited by vagal mediated acetylcholine inhibition of macrophages. Inflammatory cytokines can activate the hypothalamic–pituitary–adrenal axis releasing glucocorticoids and limiting inflammation. Parasympathetic activity is thus a regulator of inflammation^39^, and reduced parasympathetic activity will lead to increased inflammatory activity in the body. This may be one of the mechanisms that connect hyperglycemia to the inflammatory process. LGI is believed to lead to increased levels of cytokines promoting cell death by pyroptosis^4^. In this study we find associations in the whole study population between LGI with both variables related to glucose metabolism (FPG, insulin and HbA1c) and HRV variables. However, the strengths of associations between LGI and HRV varied in the subgroups. Decreased HRV and increased HR have been associated with inflammation in the Copenhagen Holter Study^21,22^, which was verified by subsequent studies^43^.

Notable, parasympathetic dysfunction is an independent negative prognostic predictor in patients with heart disease and is associated with increased cardiovascular morbidity and mortality^44^.

#### Parasympathetic tone and heart rate

Reduced parasympathetic tone results in poor heart rate control and a higher resting heart rate. A higher heart rate promotes atherosclerosis^45^. The Framingham study showed that a higher heart rate was related to cardiovascular death and morbidity independently of cardiovascular risk factors^40^. These findings have been validated in epidemiological studies^46,47^. Among patients with heart failure, parasympathetic dysfunction has been associated with a higher mortality rate^48^. This has generated the hypothesis that patients with parasympathetic dysfunction can be treated with vagus nerve stimulation to improve clinical outcomes^49^. In animal models, vagal nerve stimulation has shown significant improvement in survival among rats with chronic heart failure and diminished cardiac vagal activity^50^. Application of vagus nerve stimulation in human subjects have showed varied results. Some studies have shown an improvement in survival in people with congestive heart failure^46,48^, while other studies have shown no benefit on survival, but a significant improvement in NYHA classification and quality of life^51^. Although there is discrepancy in the results for vagal nerve stimulation it may be of great benefit in the right settings and among the right patient groups. Furthermore, stimulation site and properties of the electric current is important for the efficacy, which may be the reason for the varied results^52^.

Our findings may suggest that treating postprandial hyperglycemia can hinder or modify autonomic imbalance, however future studies are needed to answer this question. Reduction of postprandial hyperglycemia and HbA1c can be achieved with a carbohydrate-reduced high-protein diets improving β-cell function and this may be a viable solution^53,54^. Future studies should evaluate whether lifestyle changes or medical interventions aiming at reducing postprandial hyperglycemia can improve HRV and autonomic function among people with pre-diabetes. Such findings could lead to attention on earlier and more aggressive treatment of people with impaired glucose metabolism.

A limitation was that participants in this study were middle-aged and elderly Caucasian people with no apparent heart disease except hypertension. All participants with T2DM in this study were either newly diagnosed or well-controlled and, therefore, our findings may not apply to people with a longer duration of T2DM or less well-controlled diabetes. Data on frequency domain measurements of HRV was not available in this cohort. Frequency domain measurements have been associated with both pre-diabetes and T2DM^9^.

## Conclusions

In people with diabetes and pre-diabetes, increasing levels of HbA1c and age may be main factors related to impaired parasympathetic function. Markers of low-grade inflammation may not attenuate the association between impaired glucose metabolism and parasympathetic activation. It suggests that an improvement in glycemic control including postprandial hyperglycemia has clinical implications in people with prediabetes or manifest diabetes to improve parasympathetic function and related co-morbidities.

## Data Availability

The datasets used and/or analyzed during the current study are available from the corresponding author on reasonable request.

## Abbreviations

CAN: Cardiac autonomic neuropathy
FPG: Fasting plasma glucose
HRV: Heart rate variability
LGI: Low-grade inflammation
NGM: Normal glucose metabolism
RMSSD: Square root of the mean squared differences of successive NN intervals
SDNN: Standard deviation of normal-normal beats
T2DM: Type 2 diabetes mellitus
